# Evidence for a direct effect of the autonomic nervous system on intestinal epithelial stem cell proliferation

**DOI:** 10.14814/phy2.13745

**Published:** 2018-06-21

**Authors:** Elizabeth A. Davis, Weinan Zhou, Megan J. Dailey

**Affiliations:** ^1^ Neuroscience Program University of Illinois at Urbana‐Champaign Urbana Illinois; ^2^ Department of Animal Sciences University of Illinois at Urbana‐Champaign Urbana Illinois

**Keywords:** Autonomic, intestinal epithelium, stem cell

## Abstract

The sympathetic (SNS) and parasympathetic (PNS) branches of the autonomic nervous system have been implicated in the modulation of the renewal of many tissues, including the intestinal epithelium. However, it is not known whether these mechanisms are direct, requiring an interaction between autonomic neurotransmitters and receptors on proliferating epithelial cells. To evaluate the existence of a molecular framework for a direct effect of the SNS or PNS on intestinal epithelial renewal, we measured gene expression for the main autonomic neurotransmitter receptors in this tissue. We separately evaluated intestinal epithelial regions comprised of the stem, progenitor, and mature cells, which allowed us to investigate the distinct contributions of each cell population to this proposed autonomic effect. Notably, we found that the stem cells expressed the receptors for the SNS‐associated alpha2A adrenoreceptor and the PNS‐associated muscarinic acetylcholine receptors (M1 and M3). In a separate experiment, we found that the application of norepinephrine or acetylcholine decreases the expression of cyclin D1, a gene necessary for cell cycle progression, in intestinal epithelial organoids compared with controls (*P* < 0.05). Together, these results provide evidence of a direct mechanism for the autonomic nervous system influence on intestinal epithelial stem cell proliferation.

## Introduction

**Figure 1:**
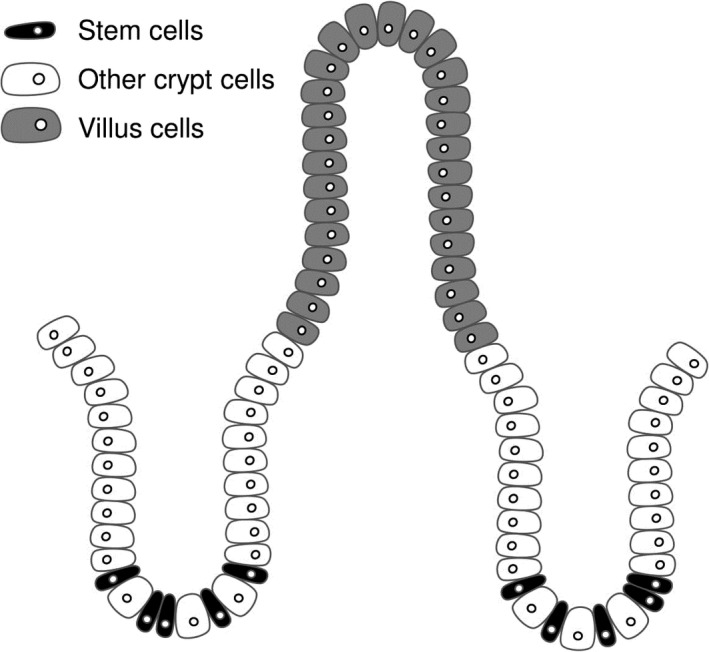
Intestinal epithelial morphology. Stem cells (black) reside at the base of the crypt. Other crypt cells (white) are primarily comprised of transit amplifying (TA) progenitor cells. The stem cells and TA cells proliferate to produce mature intestinal epithelial cells (gray), which reside in the villus and serve the major functions of the intestinal epithelium.

The intestinal epithelium is critical for nutrient absorption, hormone release, and immune barrier function. In order to maintain proper tissue function, intestinal epithelial cells undergo rapid turnover, a process that is driven by proliferating intestinal epithelial stem cells in the crypt (Barker et al. 2007). The stem cells divide to produce transit amplifying (TA) progenitor cells, which proliferate and differentiate as they migrate up the crypt. Once they reach the base of the villus, these cells are fully differentiated, and will serve their mature functions until eventually undergoing apoptosis at the villus tip (Fig. 1). Both branches of the autonomic nervous system (ANS) have been implicated in the control of this turnover process, as denervation (surgical or chemical) of either sympathetic (SNS) or parasympathetic (PNS) nerves to the intestine alters intestinal epithelial cell proliferation (Tutton and Helme 1974; Musso et al. 1975; Tsibulevskii and Orlova 1976; Lachat and Goncalves 1978; Kennedy et al. 1983; Callaghan 1991). However, it is not known if these changes occur via direct or indirect mechanisms. Previous studies may have detected indirect effects on proliferation due to postdenervation‐induced changes in food intake, inflammation, or other factors that can alter intestinal epithelial cell proliferation (Dailey 2014; Slater et al. 2017). In contrast, a direct effect could potentially be driven by autonomic neurotransmitters binding to receptors localized on proliferating cells of the intestinal epithelium. SNS and PNS neurotransmitter receptors are expressed by cells of the intestinal epithelium (Valet et al. 1993; Greig and Cowles 2017) and mediate various epithelial functions (e.g., fluid transport and hormone release) (Greenwood et al. 1987; Rocca and Brubaker 1999), but whether the stem cells or TA cells express these receptors and mediate changes in the cell cycle has not been investigated. Because a direct effect of the SNS and PNS has been shown to alter the regeneration of other tissues [e.g., liver (Cruise et al. 1985; Oben et al. 2003a)], we postulate that the SNS and PNS could modulate intestinal epithelial regeneration through direct control of the cell cycle downstream of neurotransmitter receptors.

To determine if the main neurotransmitters for the SNS and PNS, norepinephrine (NE), and acetylcholine (ACh), can mediate a direct action on the proliferating cells of the intestinal epithelium, we evaluated gene expression for the alpha2A adrenoreceptor (*Adra2a*) and the muscarinic acetylcholine receptors M1‐M5 (*Chrm1‐5,* also known as cholinergic receptor muscarinic 1–5), which were selected due to previous studies localizing them within the intestine and implicating them in control of proliferation (Valet et al. 1993; Schaak et al. 2000; Greig and Cowles 2017). We analyzed the expression of these receptors in the two major regions of the intestinal epithelium, the crypts (where the proliferating cells reside) and the villi (where the mature cells reside) in mice. Then, we further analyzed whether crypt cell receptor expression occurred in the stem cells or the other nonstem crypt cells (the vast majority of which are the TA cells). In addition, to investigate the presence of the molecular framework for the autonomic nervous system to influence intestinal epithelial turnover, we also investigated whether NE or ACh could induce changes in the cell cycle. We used intestinal epithelial organoids grown in vitro to measure the response of NE or ACh on the expression of cyclin D1 (*Ccnd1*), which is critical for the progression of the cell cycle from G1 to the S phase and remains at high levels until the end of mitosis. Both alpha2A adrenoreceptor and muscarinic acetylcholine receptor signaling have been linked to changes in cyclin D1 in other tissues, resulting in changes in the cell cycle and/or proliferation (Arredondo et al. 2003; Karkoulias and Flordellis 2007; Braga et al. 2013; Peng et al. 2013). Thus, these experiments begin to describe the molecular components and physiology through which a direct ANS control of stem cell proliferation and tissue regeneration may occur.

## Methods

### Ethical approval

All procedures were approved by the Institutional Animal Care and Use Committee at the University of Illinois at Urbana‐Champaign, which operates under the Association for Assessment and Accreditation of Laboratory Animal Care International guidelines.

### Animals

Adult male Lgr5‐GFP (B6.129P2‐Lgr5tm1(cre/ERT2)Cle/J; *n* = 3 for receptor expression experiment) and wild type (C57BL/6J; *n* = 3 for the receptor expression experiment and *n* = 3 for the proliferation‐related gene expression experiment) mice were used (Jackson Laboratory, Bar Harbor, ME). These transgenic animals were selected for their global and constitutive expression of a green fluorescent protein tag on the intestinal epithelial stem cell marker Lgr5 (Barker et al. 2007). A power analysis (G*Power 3.1.9.2) based on previously published and our preliminary data investigating mRNA expression differences between IESCs and other crypt cells show that to achieve a power = 0.8 and a type I error of 0.05, an *n* = 3 mice per group are needed (Mustata et al. 2011; Akcora et al. 2013; Tsai et al. 2014; Kechele et al. 2017; Zhou et al. 2018). Animals were single housed in shoebox cages and maintained with ad libitum access to tap water and laboratory chow (Teklad 22/5, Envigo, Madison, WI) on a 12:12 light:dark cycle (lights on 0700) in a climate‐controlled room (temperature = 21 ± 2°C and humidity = 50 ± 10%).

### Isolation of small intestinal crypts and villi

Small intestinal crypts were isolated as previously described (Sato and Clevers 2013; Zhou et al. 2018). Briefly, the animals were anesthetized under 3% isoflurane at 1.5 L/min in an anesthesia induction chamber and then decapitated. The entire small intestine was harvested, opened longitudinally and washed with cold 1× PBS to remove luminal contents. The villi were scraped off with a coverslip. Villi from the wild‐type mice (biological replicates, *n* = 3) were collected into a falcon tube, and centrifuged at 300*g* at 4°C for 5 min. The supernatant was removed and the pellet containing the villus cells was immediately stored at −80°C to await processing. The remaining intestinal tissue was cut into 2–4 mm pieces with scissors and washed 5–10 times with cold 1× PBS until the supernatant was almost clear. Tissue fragments were incubated with 2 mmol/L EDTA (Fisher Scientific, Pittsburgh, PA) and gently rocked at 4°C for 30 min. After removal of EDTA, tissue fragments were washed with 1× PBS 3 times. The supernatant was then collected and passed through a 70‐*μ*m cell strainer (Corning, Corning, NY) and centrifuged at 300*g* at 4°C for 5 min. The cell pellet was resuspended with basal culture medium [Advanced DMEM/F‐12 Medium (Gibco, Grand Island, NY) containing 2 mmol/L GlutaMax (Gibco, Grand Island, NY), 10 mmol/L HEPES (Gibco, Grand Island, NY) and 100 U/mL Penicillin‐Streptomycin (Gibco, Grand Island, NY)] and centrifuged at 300*g* 4°C for 5 min. The supernatant was removed. The isolated crypts from the wild type mice (biological replicates, *n* = 3) were immediately stored at −80°C to await processing. The isolated crypts from the Lgr5‐GFP mice (biological replicates, *n* = 3) were used for intestinal epithelial stem cell isolation (see Isolation of IESCs). Both sets of samples were then processed to evaluate gene expression (see Quantitative real time polymerase chain reaction). Isolated crypts from the second set of wild‐type mice were grown into organoids (see Organoid growth).

### Isolation of IESCs

Isolated crypts from Lgr5‐GFP mice were resuspended with single cell dissociation medium [basal culture medium containing 1× N2 (Gibco, Grand Island, NY), 1× B27 (Gibco, Grand Island, NY) and 10 *μ*mol/L Y‐27632 (Sigma‐Aldrich, St. Louis, MO)] at 37°C for 40 min. During incubation, the cell suspension was resuspended every 10 min. Dissociated cells were passed through a 40‐*μ*m cell strainer (pluriSelect, Leipzig, Germany), followed by a 20‐*μ*m m cell strainer (pluriSelect, Leipzig, Germany) and centrifuged at 300*g* at 4°C for 5 min. Single, live IESCs were sorted as GFP^high^ (stem cells) or GFP^low^ (other crypt cells) by fluorescence‐activated cell sorting (FACS) with a BD FACS ARIA II sorter into single‐cell dissociation medium as previously defined cell populations (Sato and Clevers 2013). Dead cells were excluded from the FACS with the viability dye propidium iodide (Invitrogen, Carlsbad, CA). Sorted cells were centrifuged at 300*g* 4°C for 5 min, supernatants were removed, and the pellet was immediately stored at −80°C to await processing for qPCR (see Quantitative real‐time polymerase chain reaction).

### Organoid growth

After the final centrifugation in the crypt isolation process, the supernatant was removed and crypts were re‐suspended in Matrigel (Corning Inc., Corning, NY) and plated in triplicate (three technical replicates (i.e., wells) per treatment from each biological replicate) onto a prewarmed 24‐well plate. Matrigel‐crypt mix was applied to the center of each well and then allowed to solidify for 10 min in a 37°C incubator. Once the Matrigel solidified, 500 *μ*L of IntestiCult Organoid Growth Medium (StemCell Technologies, Cambridge, MA) was added per well. Media were changed after 4 days of growth. After 5 days of growth, organoids received one of 3 treatments: 1 *μ*mol/L norepinephrine (A0937, Sigma Aldrich, St. Louis, MO), 1 *μ*mol/L acetylcholine (A2261, Sigma Aldrich, St. Louis, MO), or a vehicle control. The physiological neurotransmitters of the ANS were used instead of pharmacological agents targeting specific autonomic neurotransmitter receptors in order to most closely mimic the in vivo physiology of this system. Doses were chosen based on other studies demonstrating NE‐ or ACh‐induced proliferation in other cell types in vitro (Cruise et al. 1985; Oben et al. 2003a,b; Liu et al. 2014, 2017). Twenty‐four hours after treatment administration, Matrigel was dissolved using 1 mL QIAzol (QIAGEN; Germantown, MD), the cells vortexed, and stored at −80°C to await processing (see Quantitative real time polymerase chain reaction).

### Quantitative real‐time polymerase chain reaction (qPCR)

Total RNA was extracted from samples using RNeasy Plus Universal Mini Kit (Qiagen, Hilden, Germany). RNA was reverse transcribed to cDNA using QuantiTect Reverse Transcription Kit (Qiagen). qPCR was performed with TaqMan Universal PCR Master Mix (Applied Biosystems, Foster City, CA) and TaqMan probes (Applied Biosystems, Foster City, CA) using Applied Biosystems QuantStudio™ 7 Flex Real‐Time PCR System. Negative reverse‐transcribed samples were generated and all reactions were carried out in triplicate. Technical triplicates were generated from each biological sample in the autonomic neurotransmitter gene expression experiment, and from each cell culture well in the experiment using organoids. The following TaqMan probes were used: *Adra2a*: Mm00845383_s1, *Chrm1*: Mm00432509_s1, *Chrm2*: Mm01701855_s1, *Chrm3*: Mm00446300_s1, *Chrm4:* Mm00432514_s1*, Chrm5:* Mm01701883_s1, *Ccnd1:* Mm00432359_m1, *Gapdh*: Mm99999915_g1. To determine relative expression values, the 2^−ΔΔCt^ method was used, where triplicate Ct values for each sample were averaged and subtracted from those derived from GAPDH.

### Confirmation of compliance

The investigators understand the ethical principles under which *Experimental Physiology* operates. This work complies with the animal ethics checklist outlined in ‘Principles and Standards for Reporting Animal Experiments in *Experimental Physiology*’.

### Data analysis

All variables were analyzed using Number Crunching Statistical Software (NCSS LLC, Kaysville, UT). Data were expressed as fold change compared to control, and are expressed as mean fold change ± SD. Two‐tailed independent two‐sample Student's t‐tests were used to determine differences between: (1) *Adra2a* expression in villi versus crypts (2) *Chrm1* expression in villi versus crypts (3) *Chrm3* expression in villi versus crypts (4) *Chrm4* expression in villi versus crypts (5) *Adra2a* expression in stem cells versus other crypt cells, (6) *Chrm1* expression in stem cells versus other crypt cells (7) *Chrm3* expression in stem cells versus other crypt cells, (8) *Ccnd1* expression between NE treatment and control, and (9) *Ccnd1* expression between ACh treatment and control. Assumptions of normality, homogeneity of variance, and independence were met. No inclusion or exclusion criteria were applied to the datasets. Differences were considered to be statistically significant at *P* < 0.05.

## Results

### Autonomic neurotransmitter receptor expression in the intestinal epithelial crypts and villi

**Figure 2:**
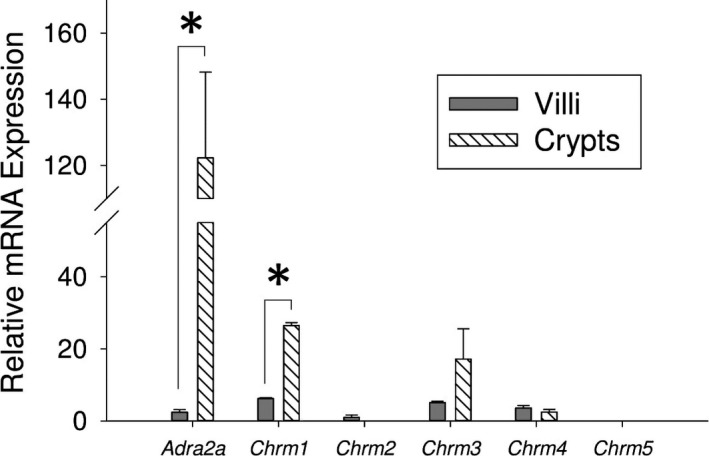
Expression of autonomic neurotransmitter receptor genes in villus and crypt cells. Relative mRNA expression of *Adra2a*,* Chrm1*,* Chrm2*,* Chrm3*,* Chrm4*, and *Chrm5* in the intestinal epithelial villi and crypts (*n* = 3, *P < 0.05).

We found that the crypts expressed *Adra2a* (122.3‐fold ± 45.8), *Chrm1* (26.4‐fold ± 0.8), *Chrm3* (17.2‐fold ± 8.4), and *Chrm4* (2.4‐fold ± 0.7), but there was no detectable expression of *Chrm2* nor *Chrm5* (Fig. 2). We found that the villi expressed *Adra2a* (2.4‐fold ± 0.7), *Chrm1* (6.2‐fold ± 0.2), *Chrm2* (1.0‐fold ± 0.7), *Chrm3* (5.1‐fold ± 0.4), and *Chrm4* (3.6‐fold ± 0.7), but there was no detectable expression of *Chrm5* (Fig. 2). *Adra2a* was expressed at higher levels in the crypt compared with the villi (*P* = 0.015; crypt: 122.3‐fold ± 45.8 vs. villi: 2.4‐fold ± 0.7, estimated difference: 119.9‐fold, 95% confidence limits of difference: 78.4 ≤ *d *≤ 161.5; Fig. 2). Similarly, *Chrm1* was also expressed at higher levels in the crypt compared with the villi (*P* = 2.0 × 10^−6^; crypt: 26.4‐fold ± 0.8‐fold vs. villi: 6.2‐fold ± 0.2 estimated difference: 20.2‐fold, 95% confidence limits of the difference: 18.9 ≤ *d *≤ 21.6, Fig. 2).

### Autonomic neurotransmitter receptor expression in intestinal epithelial stem cells and other crypt cells

**Figure 3:**
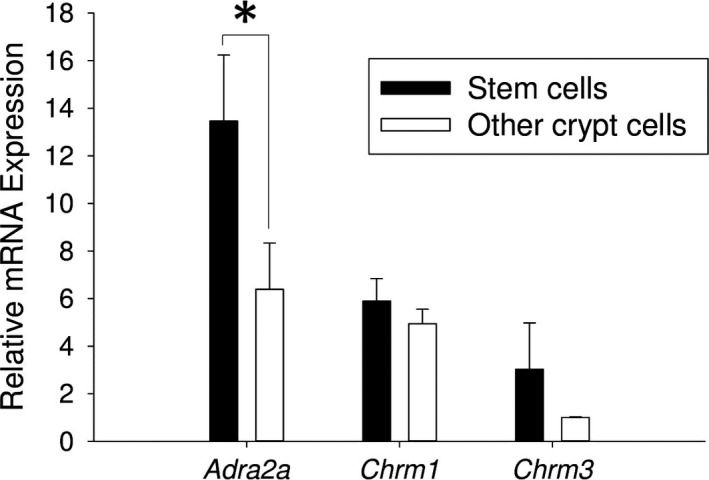
Expression of autonomic neurotransmitter receptor genes in stem cells and other crypt cells. Relative mRNA expression of *Adra2a*,* Chrm1*, and *Chrm3* in the intestinal epithelial stem cells and other crypt cells (*n* = 3, *P < 0.05).

We found that the stem cells expressed *Adra2a* (13.5‐fold ± 2.8), *Chrm1* (5.9‐fold ± 0.9), and *Chrm3* (3.0‐fold ± 2.0) (Fig. 3). We found that the other crypt cells also expressed *Adra2a* (6.4‐fold ± 2.0), *Chrm1* (4.9‐fold ± 0.6), and *Chrm3* (1.0‐fold ± 0.03) (Fig. 3). *Adra2a* was expressed in the stem cells to a higher level than the other crypt cells (*P* = 0.022; stem cells: 13.5‐fold ± 2.8 vs. other crypt cells: 6.4‐fold ± 2.0, estimated difference: 7.1‐fold, 95% confidence limits of the difference: 1.6 ≤ *d* ≤ 12.5, Fig. 3).

### Effect of autonomic neurotransmitters on expression of a proliferation‐related gene in intestinal epithelial organoids

**Figure 4:**
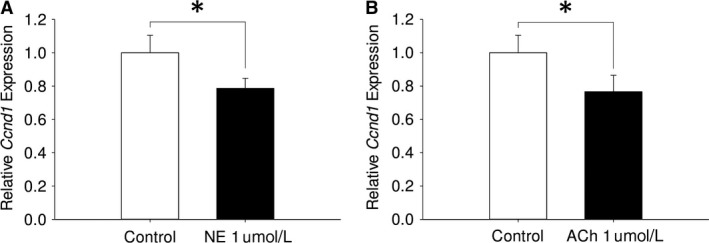
Effect of autonomic neurotransmitters on expression of a proliferation‐related gene in intestinal epithelial organoids. (A) Effect of NE on relative *Ccnd1* mRNA expression in intestinal epithelial organoids compared with control (*n* = 3; *P < 0.05). (B) Effect of ACh on relative *Ccnd1* mRNA expression in intestinal epithelial organoids compared with control (*n* = 3; *P < 0.05).

NE decreased relative mRNA expression of *Ccnd1* in intestinal epithelial organoids compared with control (*P* = 0.037; NE: 0.78‐fold ± 0.06 vs. control: 1.0‐fold ± 0.10, estimated difference: 0.21‐fold, 95% confidence limits of the difference: 0.02 ≤ *d* ≤ 0.41, Fig. 4A). ACh decreased expression of *Ccnd1* intestinal epithelial organoids compared with control (*P* = 0.047; ACh: 0.77‐fold ± 0.10 vs. control: 1.0‐fold ± 0.10, estimated difference: 0.23‐fold, 95% confidence limits of the difference: 0.003 ≤ *d* ≤ 0.36, Fig. 4B).

## Discussion

This study determined that the SNS and PNS autonomic neurotransmitter receptors are expressed in the villus, crypt, and stem cells of the intestinal epithelium. Notably, *Adra2a*,* Chrm1*, and *Chrm3* were expressed in both populations of proliferating cells, the stem cells and the TA cells. In addition, we found that application of NE or ACh decreased the expression of cyclin D1 in intestinal epithelial organoids. Together, these findings suggest that both the SNS and PNS may be capable of directly influencing intestinal epithelial cell proliferation by a direct modulation of the stem cells.

SNS and PNS nerves come in close contact with the intestinal epithelium, which supports the idea of a direct autonomic effect on intestinal epithelial cell proliferation and tissue renewal (Gabella and Costa 1968; Bohorquez et al. 2015). NE and ACh released from these terminals may bind to receptors on the stem cells and/or the TA cells to affect proliferation. Both NE and ACh have been shown to directly alter proliferation in other cell types, mediated through a variety of adrenoreceptor and muscarinic receptor subtypes (Cruise et al. 1985; Geloen et al. 1988; Bronnikov et al. 1992; Oben et al. 2003a,b; Liu et al. 2014, 2017; Sloniecka et al. 2015; Morgan et al. 2016). However, in contrast to other organ systems, the gastrointestinal (GI) tract is innervated by the enteric nervous system (ENS). The ENS makes synaptic contacts with both the SNS and PNS, but also provides intrinsic neural control of gastrointestinal functions independent of extrinsic innervation. Due to input from multiple neural systems, the source of neurotransmitters effecting changes in intestinal epithelial cells must be considered. All noradrenergic innervation of the GI tract is sympathetic, as the ENS does not utilize NE as a neurotransmitter (McConalogue and Furness 1994). Thus, our results demonstrating that NE alters expression of cyclin D1 can be attributed exclusively to the SNS, without participation of enteric neurotransmission. In contrast, since ACh is both a PNS and ENS neurotransmitter, it is not possible to elucidate the effect of PNS versus ENS sources of ACh in the present experiment alone. However, considering PNS denervation alters intestinal epithelial cell proliferation despite enteric circuitry remaining intact (Musso et al. 1975; Tsibulevskii and Orlova 1976; Lachat and Goncalves 1978; Callaghan 1991), it is plausible that the PNS participates in mediating intestinal epithelial cell proliferation via ACh signaling. Thus, our results support the narrative that the intestinal epithelium is among the tissues in which regeneration is under direct SNS and PNS control.

We found that both NE and ACh both decrease expression of cyclin D1 in intestinal epithelial cells in vitro. As the effects of the SNS and PNS are usually thought of as antagonistic, opposing effects of NE and ACh on cyclin D1 might be expected. However, a more nuanced description of the ANS includes cooperative function of the SNS and PNS on certain organs (e.g., iris muscles in the eye and sexual organs (Wehrwein et al. 2016)). This SNS and PNS cooperation is also seen in regard to control of cell proliferation, as NE and ACh both increase proliferation of hepatic myofibroblastic stellate cells, reflecting the dual participation of the SNS and PNS in liver regeneration after injury (Oben et al. 2003a,b). Similarly, the SNS and PNS may differentially coordinate suppression of intestinal epithelial cell proliferation in vivo. This may be a strategy for the ANS to inhibit proliferation during specific body states. When sympathetic tone is high, the SNS coordinates an overall decrease in energy and resources to the intestine (Browning and Travagli 2014; Wehrwein et al. 2016), which may subsequently limit the energy available for cell proliferation. In contrast, PNS tone is increased during digestion, which is accompanied by increased cellular metabolism and subsequent reactive oxygen species (ROS) byproducts (Granger et al. 2015). Because ROS induces DNA damage that can cause DNA synthesis errors, this may not be an ideal environment for cell proliferation (Oberreuther‐Moschner et al. 2005; Cadet and Wagner 2013). Thus, by decreasing cyclin D1 expression under conditions of exceptionally high SNS or PNS tone, the ANS may be restricting progression of the cell cycle during physiological states that are unfavorable for proliferation.

This study also revealed which autonomic neurotransmitter receptors are expressed in proliferating cell populations of the intestinal epithelium, thus identifying candidates for mediation of this proposed direct effect of the ANS on proliferation. Previous research has shown that the disruption of these receptors can modulate proliferation. Intestinal epithelial cell proliferation is increased in transgenic mice with global knockouts of M1 (*Chrm1*) or M3 (*Chrm3*) compared with wild‐type controls (Greig and Cowles 2017). In addition, proliferation is altered in a CaCo2 cell line model of intestinal epithelial cells transfected to express the alpha2A adrenoreceptor (*Adra2a*) (Schaak et al. 2000). Therefore, under homeostatic conditions, it is likely that the specific receptors we have identified on the stem cells and the TA cells are directly modulating proliferation via autonomic neurotransmitter signaling.

We can now use the results from this study as a framework to definitively demonstrate which specific proliferating cell subpopulation is driving these changes in proliferation‐related events: the stem cells, the TA cells, or a combination of both cell types. Stem cell isolation techniques used in this paper can be further employed to answer these research questions, which will add to the understanding of the interactions between somatic stem cells and the autonomic nervous system. In addition, we can also reveal the exact intracellular signaling mechanisms that are causing changes in cyclin D1 downstream of the autonomic neurotransmitter receptors. Our current results provide a foundation for in‐depth exploration of neural control of stem cells in the intestinal epithelium, and can be expanded to investigate stem cells from other tissues throughout the body. Further research on this topic is necessary to elucidate the mechanistic details and physiological function of this phenomenon.

## Conflict of Interest

None declared.
